# Application of Smart Healthcare in Comparative Analysis of Effect of Early External Fixator and Plate Internal Fixation Treatment on Postoperative Complications and Lower Limb Function Recovery of Patients With Unstable Pelvic Fracture

**DOI:** 10.3389/fpubh.2022.887123

**Published:** 2022-04-29

**Authors:** Hongwei Zhang, Fang Sun, Yao Li

**Affiliations:** ^1^Department of Emergency, The First Affiliated Hospital of Jiamusi University, Jiamusi, China; ^2^Plastic Burn Beauty Center, The First Affiliated Hospital of Jiamusi University, Jiamusi, China; ^3^Department of Orthopeadic Surgery, The First Affiliated Hospital of Jiamusi University, Jiamusi, China

**Keywords:** early external fixator, plate internal fixation, unstable pelvic fracture, postoperative complications, lower limb function recovery, smart healthcare

## Abstract

**Objective:**

To comparatively analyze the effect of early external fixator and plate internal fixation treatment on postoperative complications and lower limb function recovery of patients with unstable pelvic fracture based on smart healthcare.

**Methods:**

The clinical data of 98 patients with unstable pelvic fractures treated in our hospital from August 2018 to August 2021 were collected for retrospective analysis, and the patients were split into group A (plate internal fixation, *n* = 49) and group B (early external fixator treatment, *n* = 49) according to the surgical modalities. The time of operation, intraoperative blood loss, postoperative complications, etc. were compared between the two groups.

**Results:**

Between the two groups, group B had significantly better clinical indicators (*P* < 0.001), a greatly higher good rate of fracture reduction and postoperative Harris score (*P* < 0.05), and obviously lower VAS score and total incidence rate of postoperative complications (*P* < 0.05).

**Conclusion:**

Through the analysis based on smart healthcare, it is found that compared with plate internal fixation treatment, early external fixator treatment presents a better effect in treating patients with unstable pelvic fracture, because it is a reliable method to accelerate fracture healing, reduce postoperative complications, and improve lower limb function.

## Introduction

Pelvic fractures account for approximately 3% of all fractures. Unstable pelvic fractures are classified as high-energy injuries (1) and are often associated with other complications, with high mortality and disability rates. Conservative treatment is often selected in clinics to treat unstable pelvic fractures, but the incidence rates of complications such as hypostatic pneumonia and gastrointestinal hemorrhagic stress ulcer are higher (2), which, despite current improvements in medical technology, still affect the prognosis of patients, resulting in a severe reduction in their quality of life (QOL) (3, 4). Since the pelvic bone is not stable and relies mainly on soft tissues such as ligaments, so the key to treatment is to provide it with an environment that is mechanically stable throughout its structure, thus aiding the recovery of both the bone and soft tissues (5). With the progress of Internet technology, China proposes to develop the smart health industry and promote the deep integration of cloud computing, big data, mobile Internet, and the health services industry, under which context smart healthcare was born. Smart healthcare enables a more scientific management model of daily life and production that greatly improves the distribution and utilization efficiency of resources. Plate internal fixation is currently a common method for the clinical treatment of unstable pelvic fractures, and clinical studies (6) have found that although it has a better fixation effect, there are disadvantages such as a long time of surgery, high difficulty in surgical manipulation, long time of incision healing and more postoperative complications. The external fixation technique is mostly used in the first aid of pelvic fractures because it plays an important role in the stability and hemodynamics of pelvic fractures and saves valuable time for the management of combined injuries, and its effect has been demonstrated in some fracture types, such as femur fractures, tibial shaft fractures, and comminuted intra-articular fractures of the distal radius in children (7–9). Currently, there are fewer clinical studies on the external fixation treatment for pelvic fractures. The study, based on smart healthcare, further summarized and concluded the diagnosis and surgical treatment for fracture types to better apply the external fixation technique in pelvic fractures, and meanwhile, measured the best position and angle for external fixator screw installation according to patients' three-dimensional CT images of the pelvis before surgery, in the hope of providing greater theoretical and data support for the surgery.

## Materials and Methods

### General Data

**Figure 1:**
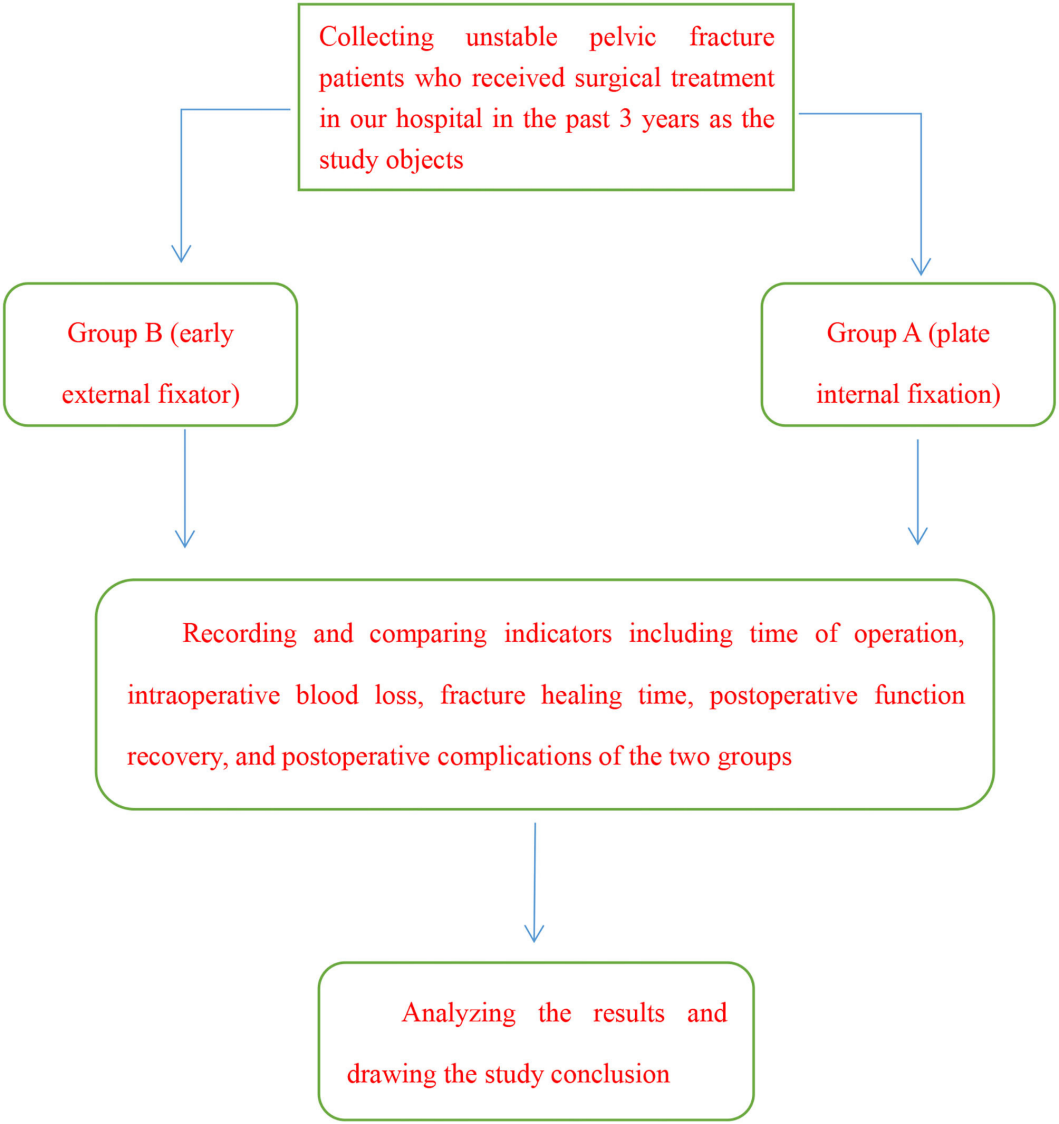
Technical rout.

The study objects were patients with unstable pelvic fracture treated in our hospital. All patients received relevant examinations after admission, see Figure 1 for the technical route. The study met the *World Medical Association Declaration of Helsinki (2013)* (10).

### Inclusion and Exclusion Criteria

Inclusion criteria. ① The images met the X-ray signs of unstable pelvic fracture; ② the time from injury to surgery ≤ 3 weeks; ③ the patients met the indications of surgical treatment; and ④ the patients had complete clinical data.

Exclusion criteria for the patients. ① Pathological or old fracture; ② complicated with severe head trauma, organic injury, or neurovascular injury; and ③ severe osteoporosis.

### Therapies

Patients in group A received internal fixation with steel plate, and the steps were as follows. General anesthesia was performed, before surgery, 0.08 mg/kg of midazolam (manufacturer: Yichang Humanwell Pharmaceutical Co., Ltd.; NMPA approval no. H20067040; specification: 2 ml: 2 mg) was administered via intramuscular injection, and 0.03 mg/kg of midazolam, 3 μg/kg of fentanyl (manufacturer: Jiangsu Nhwa Pharmaceutical Co., Ltd.; NMPA approval no. H20113509; specification: 10 ml: 0.5 mg), 1.5 mg/kg of propofol (manufacturer: Sichuan Guorui Pharmaceutical Co., Ltd.; NMPA approval no. H20040079; specification: 10 ml: 0.1 g) and 0.6 mg/kg of rocuronium (manufacturer: Guangdong Jiabo Pharmaceutical Co., Ltd.; NMPA approval no. H20183109; specification: 5 ml: 50 mg) was given *via* an intravenous drip. For patients with pure anterior ring injury such as separation of the pubic symphysis, the Pfannenstiel incision approach was adopted to expose the pubis and pubic symphysis site, the tissues inside the inguinal canal were well protected, and pelvic reduction was performed with pelvic reduction forceps or screw reduction forceps, the pelvic reconstructive plate was used for fixation, which was placed above the pubic symphysis site. For patients with fractures at the root of the superior branch of the pubis and acetabulum, an ilioinguinal approach was adopted, the femoral veins, femoral arteries, and peripheral nerves were well protected, after reduction, fixation was performed with the reconstructive plate, which was placed above the pubic branch, and C-arm X-ray was used to confirm that the screws were not in the acetabulum. For patients with pubic branch fracture at the anterior pelvic ring or mild separation of the pubic symphysis, if the pelvis was stable after reduction and fixation of the posterior ring, the method of single posterior ring fixation could be adopted (11). For patients with subluxation or luxation of the sacroiliac joint, an ilioinguinal approach was adopted to expose the front of the whole sacroiliac joint and iliac fossa, subperiosteal dissection was performed, the Kirschner wire (manufacturer: Tianjin Jinxingda Industries Co., Ltd.; model: type ZQY) was screwed in sacrum from the outside of L_5_ nerve root, and fixation was conducted with 2- to 4-hole steel plate. For patients with sacroiliac joint luxation or sacrum fracture, a posterior exposure approach was performed, the patients were in the supine position, a straight or arc-shaped incision was made along the sacroiliac joint, the gluteus maximus was separated from the attachment point, and the superior gluteal blood vessels and nerves were protected. Closed reduction was mostly adopted for the fixation of iliosacral screws, the patients were in the supine position or prone position, the C-arm system confirmed that the position, direction, and length were appropriate in 3 planes on the lateral part, pelvic inlet, and outlet, and the corresponding screws were selected for fixation of fracture and luxation (12).

Patients in group B received early external fixator treatment. After general anesthesia, the patients were in the supine position, routine disinfection and draping were conducted, and before skin incision, 1 g of tranexamic acid (manufacturer: Shandong Yijian Pharmaceutical Co., Ltd.; NMPA approval no. H20043218; specification: 10 ml: 1.0 g) was administered via intravenous infusion. The anterior inferior iliac spine was located by C-arm X-ray, the skin and subcutaneous tissues were cut open, and the lateral femoral cutaneous nerves were protected; intermuscular blunt dissection was performed to expose the anterior inferior iliac spine and place the sleeve and drill holes with a depth over 3 cm (13). After withdrawing the drill, the threaded nails 6 mm in diameter and 150 mm long were screwed in with a depth over 3 cm, and attention should be paid to avoid entering the hip joint. When the fracture reduction was satisfied, according to the pelvic 3D-CT image before surgery, the best screw installation position and angle of the external fixator were measured, the connecting rod was installed and the nuts were fixed, and then routine suture was performed.

### Observation Indicators

Various perioperative indicators of patients in the two groups were recorded, including time of surgery, intraoperative blood loss, fracture healing time, and total length of the incision.

During postoperative follow-up, reduction evaluation was conducted according to the pelvic imaging data and by Matta's criteria for pelvic ring fractures (14), with displacements of less than 4 mm indicating excellent, 4–10 mm indicating good, 10–20 mm indicating fair, and more than 20 mm indicating poor. The scale could be used to evaluate the quality of fracture reduction, and the good rate = (number of excellent cases + number of good cases + number of fair cases) / total number of cases × 100%.

The patients' hip joint recovery after surgery was assessed by the Harris Hip Scale (HHS) (15), which covered four domains, function, pain, absence of deformity, and range of motion. The maximum score was 100 points, with more than 90 points indicating excellent, 80–90 points indicating good, 70–79 points indicating fair, and less than 70 points indicating poor.

The degree of postoperative pain was assessed by the Visual Analog Scale (VAS) (16), to be specific, a vernier slide caliper about 10 cm long was marked with 10 scales on one side and “0” (no pain) and “10” (the worst pain imaginable) at both ends. In clinical use, the side without the scales was faced to the patients for them to pick the corresponding position that best indicated their current degree of pain, and the doctor gave the score accordingly.

The occurrence of complications after-treatment of the two groups was recorded, including venous thrombosis, neural injury, and incision infection.

### Statistical Methods

In this study, the data were processed by the professional statistic software SPSS26.0 (IBM, NY, USA), the picture drawing software was GraphPad Prism 7 (GraphPad Software, San Diego, USA), the enumeration data were examined by *X*^2^ test and expressed by [n(%)], the measurement data were examined by *t*-test and expressed by Mean±SD, and differences were considered statistically significant at *P* < 0.05.

## Results

### Clinical Data

No significant between-group differences in clinical data including gender ratio, time from injury to surgery, and cause of injury were observed (*P* > 0.05), presenting comparability. See Table 1.

**Table 1:** Clinical data (*n* = 49).

**Item**	**Group A**	**Group B**	**X^**2**^/t**	**P**
Gender			0.167	0.683
Male/female	27/22	29/20		
BMI (Mean ± SD, kg/m^2^)	22.12 ± 1.24	22.43 ± 1.42	−1.169	0.245
Mean age (Mean ± SD, years)	46.61 ± 9.52	45.36 ± 11.54	0.580	0.563
Time injury to surgery (Mean ± SD, d)	6.12 ± 2.26	6.35 ± 2.53	−0.461	0.646
Tile classification			0.400	0.527
B	30 (61.22)	33 (67.35)		
C	19 (38.78)	16 (32.65)		
Cause of injury			0.699	0.873
Traffic accident	23 (46.94)	26 (53.06)		
Fall from height	10 (20.41)	8 (16.33)		
Crushing by weight	12 (24.49)	10 (20.41)		
Others	4 (8.16)	5 (10.20)		
Educational degree			0.238	0.888
College	7 (14.29)	8 (16.33)		
Middle school	28 (57.14)	29 (59.18)		
Primary school	14 (28.57)	12(24.49)		
Place of residence			0.656	0.418
Urban area	25 (51.02)	21 (42.86)		
Rural area	24 (48.98)	28 (57.14)		

### Perioperative Indicators

Various perioperative indicators were significantly better in group B than in group A (*P* < 0.001). See Table 2.

**Table 2:** Perioperative indicators [Mean ± SD].

**Group**	**n**	**Time of operation (min)**	**Intraoperative blood loss (mL)**	**Fracture healing time (d)**	**Total length of incision (cm)**
A	49	105.47 ± 18.83	102.01 ± 12.26	108.82 ± 8.71	23.05 ± 3.09
B	49	69.81 ± 8.43	75.09 ± 7.35	95.93 ± 4.47	19.11 ± 2.90
t		12.099	13.183	9.214	6.505
P		<0.001	<0.001	<0.001	<0.001

### Fracture Reduction Quality

The good rate of fracture reduction was significantly higher in group B than in group A (*P* < 0.05). See Table 3.

**Table 3:** Fracture reduction quality [n(%)].

**Group**	**n**	**Excellent**	**Good**	**Fair**	**Poor**	**Good rate**
A	49	17 (34.69%)	19 (38.78%)	5 (10.20%)	8 (16.33%)	83.67% (41/49)
B	49	23 (46.94%)	17 (34.69%)	7 (14.29%)	2 (4.08%)	95.92% (47/49)
X^2^						4.009
P						0.045

### Harris Scores

**Figure 2:**
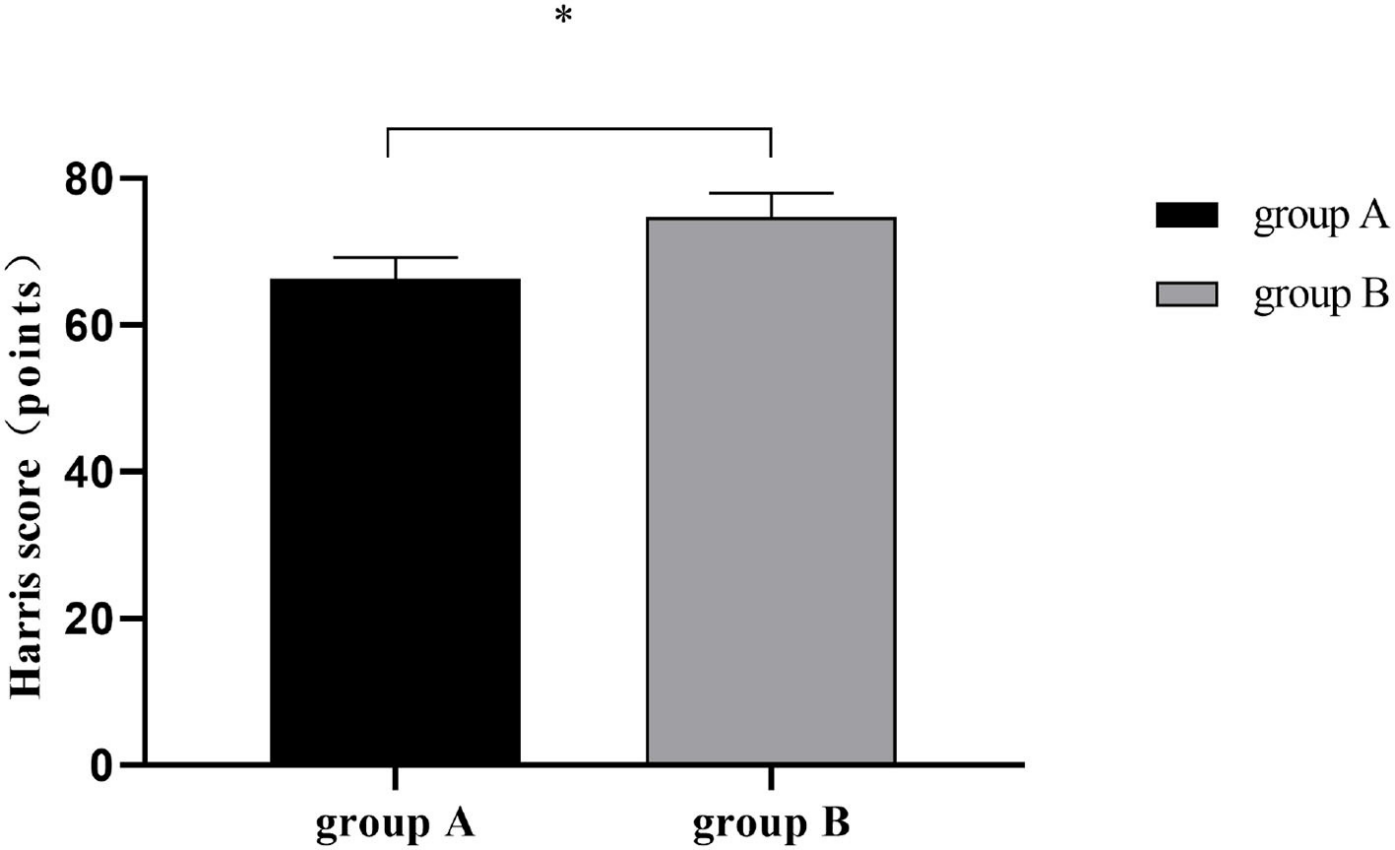
Postoperative Harris scores [Mean ± SD]. The horizontal axis denoted groups A and B, and the vertical axis denoted the Harris score (points); After surgery, the Harris scores of groups A and B were respectively (66.37 ± 2.83) and (74.78 ± 3.28); and *indicated a significant between-group difference in postoperative Harris scores (t = 13.589, *P* < 0.001).

The postoperative Harris score was remarkably higher in group B than in group A (*P* < 0.001). See Figure 2.

### VAS Scores

**Figure 3:**
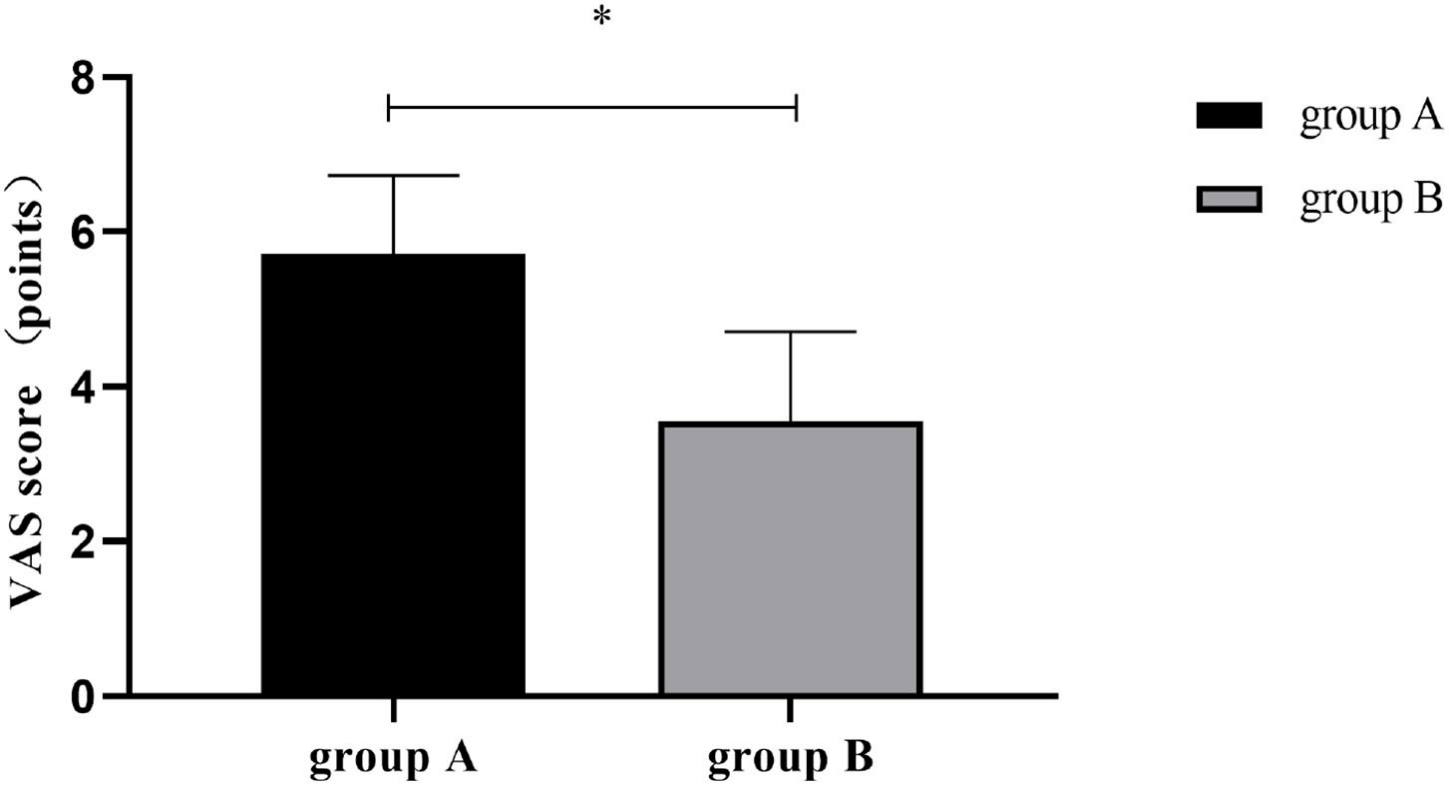
Postoperative VAS scores [Mean±SD]. The horizontal axis denoted groups A and B, and the vertical axis denoted the VAS score (points); After surgery, the VAS scores of groups A and B were respectively (5.71 ± 1.02) and (3.55 ± 1.16); and *indicated a significant between-group difference in postoperative VAS scores (t = 9.789, *P* < 0.001).

The postoperative VAS score was significantly lower in group B than in group A (*P* < 0.001). See Figure 3.

### Postoperative Complications

The total incidence rate of postoperative complications was significantly lower in group B than in group A (*P* < 0.05). See Table 4.

**Table 4:** Postoperative complications [n(%)].

**Group**	**n**	**Venous thrombosis**	**Nerve injury**	**Incision infection**	**Total incidence rate**
A	49	2 (4.08)	2 (4.08)	4 (8.16)	16.33% (8/49)
B	49	0 (0.00)	1 (2.38)	1 (2.38)	4.08% (2/49)
X^2^					4.009
P					0.045

## Discussion

Smart healthcare is a rapidly developing high-tech means of medical treatment in the era of big data, and it has gradually infiltrated into people's daily lifestyles. This technique helps people better solve problems in the field of medical health through artificial intelligence (AI) technology, big data, cloud computing, etc., and then opens up new directions in the construction of medical fields. Each patient is considered as an independent individual in smart healthcare, and multiple diagnosis and treatment modalities depending on different patient conditions can be provided, thereby promoting medical quality. Pelvic fractures are injuries resulting from high energy, which are often accompanied by multiple life-threatening injuries to other parts of the body, so the implementation of emergency trauma control and restoration of pelvic ring stabilization is essential for patient survival and treatment. Since the deformity caused by pelvic fracture usually triggers long-term dysfunction, surgical reduction and fixation need to be performed to restore the stability of the pelvis (17). Due to the complexity of pelvic structures and fracture types, surgery is often challenging, so there is currently no consensus on the surgical approach and the choice of fixation method. As a simple method of external fixation, traditional complete pelvic fixation has many advantages, such as easy operation and access, making it a common surgical procedure for the treatment of unstable pelvic fractures for a time (18). However, the poor fixation strength and fixation results of this technique make it unsuitable for some complex pelvic fractures. The previously used internal fixation treatment methods have the shortcomings such as a long time of surgery, high difficulty in surgery, long time of postoperative healing, and more postoperative complications. The external fixator technique is frequently applied in the emergency treatment of pelvic fractures and plays a key role in performing reduction, controlling bleeding, and reducing mortality (12). By drawing on previous clinical treatment experience, this study better exerts the application of external fixator technology in a pelvic fracture through a comparative analysis of the clinical effects of early external fixator and plate internal fixation in the treatment of unstable pelvic fracture, presenting a certain reference and guidance significance for the treatment of an unstable pelvic fracture.

The study results showed that the postoperative good rate of fracture reduction was significantly higher in group B than in group A (*P* < 0.05), which was due to the fact that the adjustable external fixation technique can reduce bleeding at the pelvic fracture end and stabilize the condition, providing a surgical opportunity for other combined injuries. In addition, the study also found that, compared with plate internal fixation treatment, an early external fixator can reduce intraoperative blood loss, time of surgery, and total length of incision to a certain extent, and accelerate the fracture healing time, which may be due to the fact that the external fixator treatment causes little skin and muscle damage and only needs to place screws firmly (19), and the efficacy of such easily operative procedure has been demonstrated in multiple metatarsal fractures (20). Moreover, the study results showed that the total incidence rate of postoperative complications was remarkably lower in group B than in group A (*P* < 0.05), and the reason may be that plate internal fixation uses the steel plate to stabilize the anterior and posterior pelvic ring for reconstruction, so as to restore fracture reduction and associated site function with the help of the plate. Some studies have pointed out (21, 22) that plate internal fixation, although has a good effect, can easily induce damage to soft tissues and nerve tissues and increase the chance of postoperative complications because of the large intraoperative incision and high impact on the body and some soft tissues. Whereas external fixator treatment is highly adjustable because the angulation can be adjusted by the attachment of fixators, which greatly reduces the occurrence of complications and allows the patient to turn over after surgery, alleviates decubital ulcers resulting from prolonged bed rest, and makes nursing more convenient. Meanwhile, it also can increase patient stability during handling and inspection to some extent, and then reduce secondary damage and complications (23, 24). The shortcomings of the study were the small sample size and a single source of cases. So further biomechanical study should be carried out to comprehensively observe the clinical effects of the two different fixation methods, so as to provide an evidence-based basis for the treatment of unstable pelvic fracture patients.

In conclusion, through analysis in the context of intelligent medicine, it is found that early external fixator treatment has better results in treating patients with unstable pelvic fractures. In recent years, with the promotion and improvement of technologies such as minimally invasive surgery and spine-pelvis combined fixation, various new directions have appeared in the treatment of pelvic fracture, meanwhile, with the continuous development of imaging-guided systems and the deepening research of biological models of real pelvic fracture established by computer simulation, conditions have been provided for the broadening of new ideas for clinicians, and the treatment of stable pelvic fractures will continuously develop in the aspects of ease of operation and firmness.

## Data Availability Statement

The original contributions presented in the study are included in the article/supplementary material, further inquiries can be directed to the corresponding author/s.

## Ethics Statement

The studies involving human participants were reviewed and approved by the Ethics Committee of the First Affiliated Hospital of Jiamusi University. The patients/participants provided their written informed consent to participate in this study. Written informed consent was obtained from the individual(s) for the publication of any potentially identifiable images or data included in this article.

## Author Contributions

HZ and YL: conception and design, data analysis, and interpretation. All authors: administrative support, provision of study materials or patients, collection and assembly of data, manuscript writing, and final approval of manuscript.

## Funding

This research was funded by Scientific research project of Heilongjiang Provincial Health Commission (Grant No. 2020-327).

## Conflict of Interest

The authors declare that the research was conducted in the absence of any commercial or financial relationships that could be construed as a potential conflict of interest.

## Publisher's Note

All claims expressed in this article are solely those of the authors and do not necessarily represent those of their affiliated organizations, or those of the publisher, the editors and the reviewers. Any product that may be evaluated in this article, or claim that may be made by its manufacturer, is not guaranteed or endorsed by the publisher.
